# A Novel GaN Metal-Insulator-Semiconductor High Electron Mobility Transistor Featuring Vertical Gate Structure

**DOI:** 10.3390/mi10120848

**Published:** 2019-12-05

**Authors:** Zhonghao Sun, Huolin Huang, Nan Sun, Pengcheng Tao, Cezhou Zhao, Yung C. Liang

**Affiliations:** 1 School of Optoelectronic Engineering and Instrumentation Science & School of Microelectronics, Dalian University of Technology, Dalian 116024, China; sunzhonghao@mail.dlut.edu.cn (Z.S.); dgsunnan@mail.dlut.edu.cn (N.S.); pctao@dlut.edu.cn (P.T.); 2 Department of Electrical and Electronic Engineering, Xi’an Jiaotong-Liverpool University, Suzhou 215123, China; cezhou.zhao@xjtlu.edu.cn; 3 Department of Electrical and Computer Engineering, National University of Singapore, Singapore 119260, Singapore; chii@nus.edu.sg

**Keywords:** wide-bandgap semiconductor, high electron mobility transistors, vertical gate structure, normally-off operation, gallium nitride

## Abstract

A novel structure scheme by transposing the gate channel orientation from a long horizontal one to a short vertical one is proposed and verified by technology computer-aided design (TCAD) simulations to achieve GaN-based normally-off high electron mobility transistors (HEMTs) with reduced on-resistance and improved threshold voltage. The proposed devices exhibit high threshold voltage of 3.1 V, high peak transconductance of 213 mS, and much lower on-resistance of 0.53 mΩ·cm^2^ while displaying better off-state characteristics owing to more uniform electric field distribution around the recessed gate edge in comparison to the conventional lateral HEMTs. The proposed scheme provides a new technical approach to realize high-performance normally-off HEMTs.

## 1. Introduction

Wide-bandgap GaN-based high electron mobility transistors (HEMTs) are promising candidates in the applications of high-frequency and high-power electronics owing to their superior material properties such as large bandgap, high critical breakdown field, and high-density carriers in the form of two-dimensional electron gas (2DEG) with high mobility over 2000 cm^2^/V·s [1,2,3,4,5]. Nowadays, much progress has been achieved in GaN-based HEMTs with the development of the material quality and the innovation of the device structure [6,7]. However, there are still several important issues unaddressed, among which, the normally-off operation with a large threshold voltage (V_th_) is a big concern when the chip products are pushed toward the market [8,9,10]. Several device architectures, such as p-GaN cap, barrier layer-recessed, fluorinated-gate, and cascode-connected metal-insulator-semiconductor high electron mobility transistors (MIS-HEMTs), have been reported to shift the threshold voltage to be positive [11,12,13,14,15,16]. Indeed, the device performances have been improved significantly in the past ten years. However, these normally-off HEMTs still suffer from either large on-resistance (R_on_) or low V_th_ which increases the risk of device switching failure. Basically, the enhancement of V_th_ comes at the expense of increasing the R_on_ of the devices. A trade-off, hence, has to be made between them [17]. In recent years, several novel normally-off schemes such as tri-gate, Fin-gate, and trench etching in the SiO_2_ buried layer have also been proposed and developed [18,19,20]. However, a truly effective device structure to gain both high V_th_ and low R_on_ simultaneously should be developed to resolve the mentioned issues effectively.

In this work, a novel scheme featuring a vertical short gate channel is proposed and demonstrated in AlGaN/GaN MIS-HEMTs to realize the normally-off operation. The source electrode is located at the trenched GaN bulk region with gate dielectric layer covering the side wall. The proposed vertical gate HEMT (VG-HEMT) is able to get a higher V_th_ (>3 V) and at the same time a lower R_on_ due to the short vertical channel (200–500 nm in this work). To verify this design, a conventional HEMT was fabricated and its output characteristics were utilized to extract the physical parameters for the subsequent TCAD simulations for the VG-HEMT design which finally exhibits a much higher output current and transconductance (g_m_) while displaying a better off-state electric field profile. The quantitative dependences of saturated current density and R_on_ on scattering factors in the trenched vertical gate channel were also investigated in detail in the work.

## 2. The Proposed Device and Physical Principle

**Figure 1:**
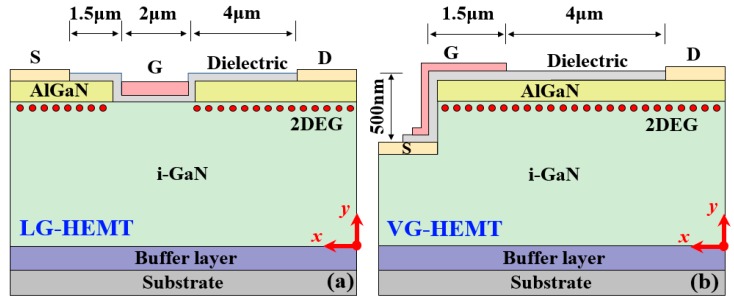
Cross-sectional schematics of (**a**) the lateral gate-recessed-high electron mobility transistors (LG-HEMT) and (**b**) the vertical gate HEMT (VG-HEMT).

Figure 1 shows the cross-sectional schematics of GaN-based HEMTs, where the same L_GD_ (distance between gate and drain) and gate length (including the gate overlap) have been employed. In the conventional lateral gate-recessed HEMTs (LG-HEMTs), as shown in Figure 1a, the length of the recessed gate is usually designed to be 1–3 μm, which is limited by the influence of the UV light diffraction in the lithography process. It is hard to get very high pattern resolution just using the common mask aligner. If the pattern resolution below 1 μm is required, a more precise and expensive lithography apparatus such as stepper has to be used. The technology processes, such as gate channel etching and metal lift-off, with resolution below 1 µm are more rigorous and challenging, and hence increase the cost. It is not easy to control the precision around few nanometers for fully etching just the thin AlGaN barrier (~20 nm), and the resulting over-etch leads to a severe degradation of 2DEG channel mobility. Therefore, an unstable V_th_ and large R_on_ are still the typical issues unaddressed till now. 

In contrast to this, the conductive 2DEG channel is removed completely by fine etching in the VG-HEMTs and the source electrode is placed at the wafer body, as shown in Figure 1b. A decent source contact can be formed by Si ion implantation beneath the source region and post-annealing treatment [21]. To address the mismatch issue, the self-aligned technology will be employed in the future to precisely adjust the source metal edge to the edge of the recessed region. To make a better isolation between gate and source, a multiple dielectric stack or a relative thick dielectric film (e.g., 30 nm) will be employed. The vertical gate on the sidewall can modulate the electric field in the vertical channel for electron accumulation and hence control the device on/off switching. Considering the large G-to-S capacitance, the VG-HEMT devices will not be used in high-frequency or microwave applications. But, for the conventional switching applications at the frequency less than 1.0 MHz, they should be able to handle. Moreover, more schemes such as increasing the dielectric thickness between the gate and source and reducing the overlap dimension of the gate electrode will be employed to reduce the capacitance. To improve the gate breakdown endurance, the bi-layer dielectric and slanted gate schemes are considered and the gate overdrive circuit will also be designed to protect the device. Compared with the long recessed-gate channel in the LG-HEMTs, the main benefits in the VG-HEMTs include a shorter gate channel originated from the low-mobility trenched region based on a simple lithography technique and greater tolerance on etching depth error. Therefore, the device is able to achieve both high V_th_ and low R_on_.

## 3. Fabrication Work and Parameter Calibration for TCAD simulation

**Figure 2:**
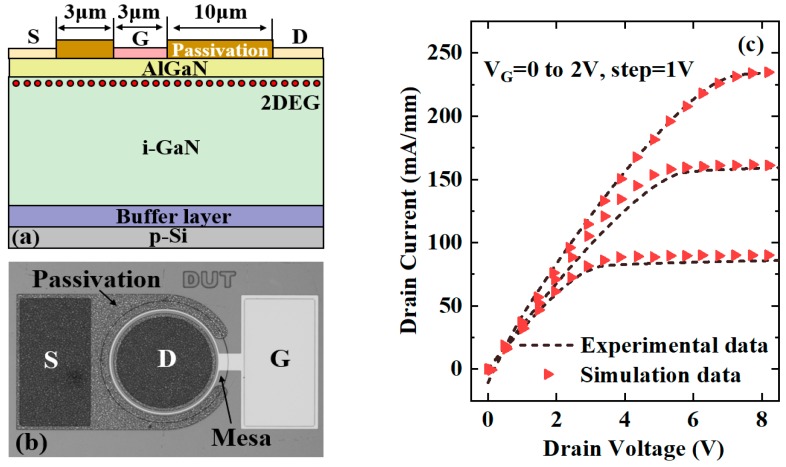
(**a**) Schematic and (**b**) microscopy image of the fabricated normally-on AlGaN/GaN HEMTs, and (**c**) comparisons of output I–V characteristics of the devices for physical parameter calibration.

The heterostructure comprising 25 nm Al_0.2_Ga_0.8_N barrier layer and 4 μm undoped GaN channel and buffer layers was grown on a 6-inch p-Si (111) substrate by metal-organic chemical vapor deposition (MOCVD) technique. The conventional GaN-based HEMTs were fabricated and characterized. The fabrication work started with the device isolation by employing Cl^-^-based plasma etching. Source and drain contacts were formed by depositing Ti/Al/Ni/Au stack using E-beam evaporation system followed by a rapid thermal annealing (RTA) at 875 °C for 30 s in N_2_ atmosphere. The gate electrode was deposited by evaporating Ni/Au alloy. Then, 300 nm SiO_2_ layer was deposited on the device surface at 300 °C by plasma enhanced chemical vapor deposition (PECVD) system for passivation purpose. Figure 2a indicates the structural schematic of the GaN HEMT. Figure 2b shows the microscopy image of the fabricated device and the I–V characteristics measured using Agilent B1505A system and benchmarked by the simulation data.

In TCAD simulations, Auger recombination, Shockley–Read–Hall (SRH) recombination, and Van Overstraeten–De Man models were used to simulate the device behaviors. To investigate the dry etching effects on the carrier mobility of gate channel in the normally-off devices, several mobility models were selected. The Arora model was employed to determine the doping-dependent mobility in the low-field case, and the transferred electron model was used to describe the effect of a transfer of electrons into an energetically higher side valley with a much larger effective mass in the high-field case [22]. The mobility degradations caused by high field and interface scattering are given by Meinerzhagen–Engl model and Lombardi model, respectively [23,24]. The comparison of simulated output I–V characteristics with the measurement data is shown in Figure 2c. The displayed I–V curves match well and the average mismatch for the current density is within 5%. This confirms the validity of the parameters used in the simulations. The physical parameters used in simulation are listed in Table 1 [3]. Especially, in the VG-HEMT, it is also of great importance to know the the mobility degradation at the etched sidewall. Thus, the effects of the etched roughness at the gate region on the device performances were investigated in detail in the next section.

## 4. Results and Discussions

**Figure 3:**
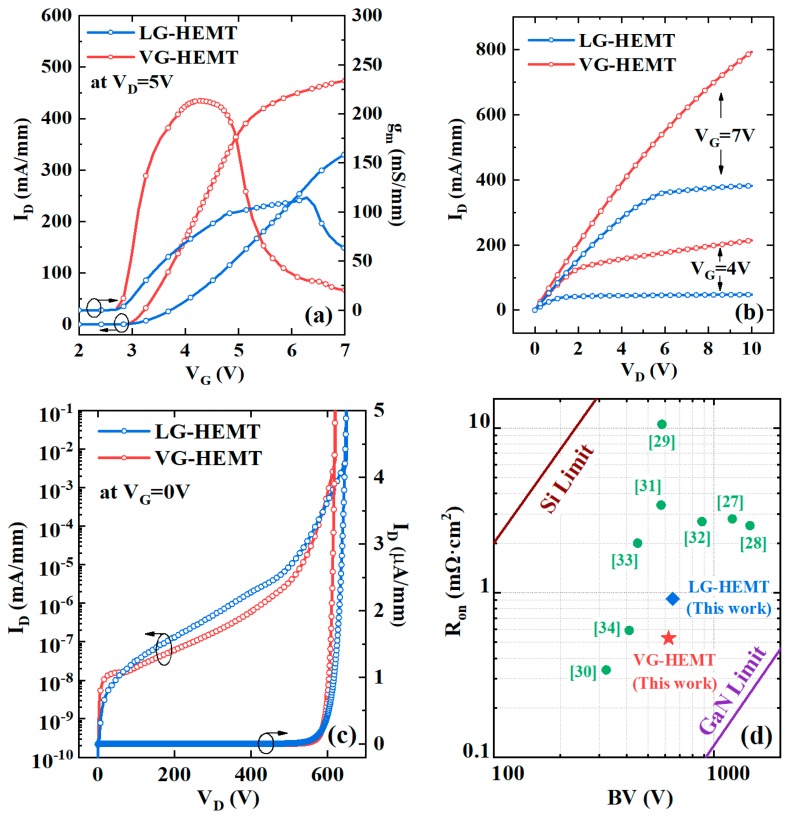
Comparisons of (**a**) transfer characteristics, (**b**) output I_D_–V_D_ curves, and (**c**) off-state breakdown characteristics between the LG-HEMT and VG-HEMT devices, and (**d**) performance comparisons among the reported normally-off HEMTs including the VG-HEMT in this work (the red star).

The output I–V characteristics of the normally-off LG-HEMT and VG-HEMT are illustrated in Figure 3. The V_th_ values are defined by extrapolating the linear section of the I_D_–V_G_ curve to the voltage axis. The normally-off operation with a positive V_th_ is realized in both devices. The VG-HEMT exhibits a V_th_ of 3.1 V and a high peak g_m_ of 213 mS/mm compared with 3.6 V and 114 mS/mm in the LG-HEMT, respectively. The similar V_th_ values in both devices are found when the influence of the polar plane is ignored considering the fact that usually the gate surface polarization might be damaged severely after a long-time plasma etching in the gate recess process and the polarization charges at the lateral surface are generally compensated by the high-density surface states, such as donor-like traps. 

Theoretically, the V_th_ model for the MIS-HEMT can be expressed in Equation (1) [25]:

(1) $$V_{th}=\frac{\varphi_{b}}{q}-\frac{\Delta E_{c}}{q}-\frac{\varphi_{f}}{q}-\frac{q\cdot \Delta Q_{it}t_{ox}}{\varepsilon_{ox}}$$

where *φ*_b_ is the barrier height for gate metal on the dielectric (3.2 eV for Ni on Si_3_N_4_), ∆E_c_ the conduction band offset between Si_3_N_4_ and GaN (1.5 eV), *φ_f_* the conduction band distance from the Fermi-level in GaN (0.2 eV), t_ox_ the dielectric thickness (20 nm), ε_ox_ the permittivity of dielectric (6.6 × 10^−13^ F/cm), and ∆*Q_it_* the net charge density at the interface (~3.4 × 10^12^ cm^−2^ in this work which is reasonable and reported widely for the III–V group GaN-based materials) [26,27]. The calculated V_th_ of the VG-HEMT is found to be 3.2 V, which is roughly consistent with the simulation data at 3.1 V. Furthermore, the slight difference of the V_th_ value between the two devices is mainly caused by the increased drain current and transconductance values in the VG-HEMT considering that the effective vertical gate length is much less than that in the LG-HEMT. The maximum current density and R_on_ are found to be 793 mA/mm and 0.53 mΩ·cm^2^ at V_G_ = 7 V in the VG-HEMT, while 381 mA/mm and 0.92 mΩ·cm^2^ in LG-HEMT, respectively. The VG-HEMT shows a higher drain current mainly owing to the saving of the L_GS_ length by placing the source electrode under the gate and a shorter gate channel originated from the low-mobility trenched region. The shorter total channel length and narrower trenched-gate originated from the structural feature of the proposed VG-HEMT result in the increase of the drain current. Furthermore, a similar off-state breakdown voltage level (606 V vs. 615 V at I_D,off_ = 1 μA/mm) is revealed between the VG-HEMT and LG-HEMT, as shown in Figure 3c. The proposed VG-HEMT exhibits even a lower drain leakage current owing to a more uniform electric field distribution. Figure 3d gives the performance comparisons with other reported normally-off HEMTs, which clearly indicates the advantage of the proposed scheme by employing a vertical short gate structure [27,28,29,30,31,32,33,34].

**Figure 4:**
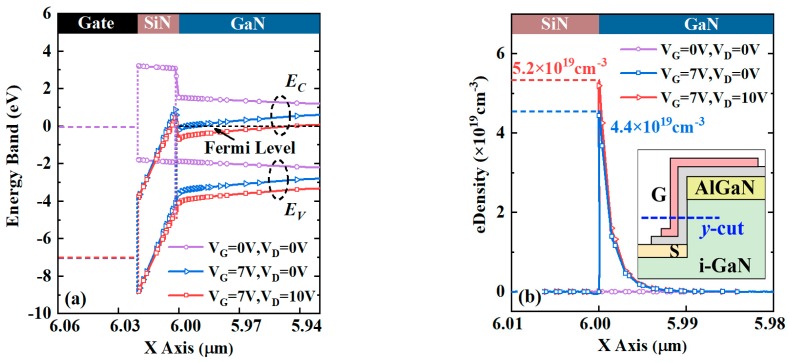

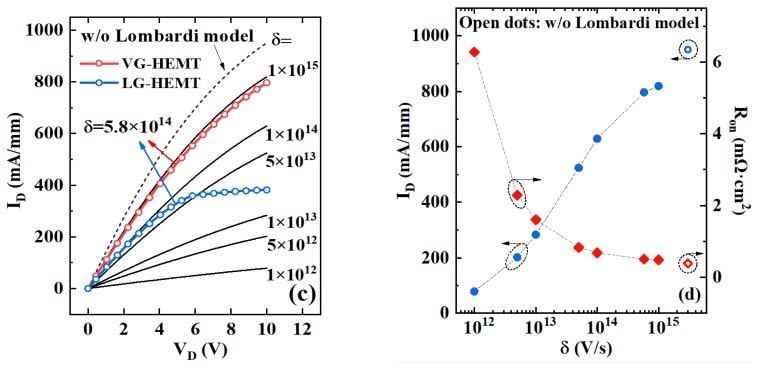
(**a**) Energy band and (**b**) electron concentration diagrams along the horizontal direction across the gate channel (“*y*-cut” marked in the inset). (**c**) I_D_–V_D_ curves affected by different δ values using Lombardi model in the VG-HEMT. (**d**) Dependences of saturated current density and R_on_ on δ values.

To further investigate the electrical behaviors of the VG-HEMT, its energy band and electron density distributions at different biases are calculated and compared, as shown in Figure 4a,b. The conduction band of vertical channel is pulled down to the Fermi level with the increasing gate and drain bias and an electron density peak is formed in the conduction channel. The surface potential of the semiconductor can be affected by the different dielectric deposition processes due to the influence of the high-density dielectric/GaN interface traps (10^12^–10^13^ cm^−2^) and the short distance to the conduction channel. As a result, the conduction band bending occurs at the interface, as shown in Figure 4a. The concentration of the accumulated electrons is given by Equation (2) [35]:

(2) $$n_{e}=\frac{C_{ox}}{q}\cdot \left(V_{g}-\frac{\varphi_{b}-\varphi_{it}-\varphi_{f}-\Delta E_{c}}{q}\right)$$

where C_ox_ is the capacitance of the gate dielectric (3.32 × 10^−7^ F/cm^−2^), V_g_ the applied gate voltage, and *φ_it_* the impact on the surface potential caused by the interface electrons. Using the ∆*Q_it_* value of 3.4 × 10^12^ cm^−2^, the calculated peak *n_e_* at V_g_ = 7 V is 7.8 × 10^12^ cm^−2^, which is approximately consistent with the data of 7.2 × 10^12^ cm^−3^ for the blue line after integral calculation in Figure 4b. 

Furthermore, the physical mechanisms of output current conduction in the VG-HEMT were analyzed. The I_D_–V_D_ characteristics of these two devices are compared because they have similar electrode distance L_GD_ and gate length L_G_. A decent source contact technology will make it easier to obtain a low source resistance and minimize the effect on the on-resistance. In this case, the surface roughness at the etched gate region becomes the major factor that influences the on-resistance of the device. The mobility degradation in the etched vertical channel is strongly dependent on several scattering factors. The sidewall channel mobility *μ* is modeled by the following expression based on Lombardi model [36]:

(3) $$\frac{1}{\mu }=\frac{1}{\mu_{\mathrm{ac}}}+\frac{1}{\mu_{b}}+\frac{1}{\mu_{\mathrm{sr}}}$$

where *μ*_ac_ is the mobility contribution by acoustic phonon scattering, *μ*_b_ the carrier mobility in bulk, and *μ*_sr_ the mobility contribution by surface roughness scattering. In the simulations, *μ*_ac_ was determined to be 818 cm^2^/(V·s) using the equation *μ*_ac_ = (BT/*E*_eff_ + C/*E*_eff_^(1/3)^)·(1/T) [24], where *E*_eff_ is the effective field controlled by the channel charge, T (= 300 K) is the temperature, and B (= 9 × 10^7^ cm/s) and C (= 5.8 × 10^2^ cm^5/3^·V^–2/3^·s^–1^) are the fitting parameters, respectively [37].The *μ*_b_ value of 803 cm^2^/(V·s) is extracted from the calibration data. Considering that *μ*_ac_ and *μ*_b_ are almost constant for a technically mature GaN wafer at a certain temperature, *μ* in the vertical channel is mainly modulated by *μ*_sr_, which can be expressed as [33]:

(4) $$\mu_{\mathrm{sr}}=\frac{\delta }{E_{\mathrm{eff}}^{\alpha }}$$

where *α* is determined by the experimental fitting. And δ is inversely proportional to the root mean square (RMS) value of the surface roughness, and is the main factor resulting in mobility degradation at the etched surface. The *μ*_sr_ value was then determined to be 1114 cm^2^/(V·s) by employing the parameters extracted from the simulation. Therefore, the mobility value of 297 cm^2^/(V·s) was achieved for the sidewall channel in VG-HEMT. The quantitative effects of δ values on the drain current density and R_on_ are shown in Figure 4c,d, taking into account the effect from the roughness scattering [37]. The VG-HEMT exhibits much better current characteristics owing to the less scattering amount in the shorter gate channel, as shown in Figure 4c. Moreover, to further investigate the effect of etching damage on the output characteristic, as well as distinguish the specific damage between the sidewall and surface, more experiments will be carried out in future work. 

**Figure 5:**
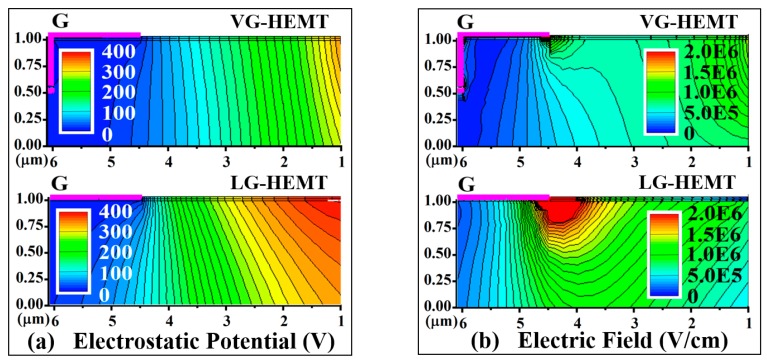

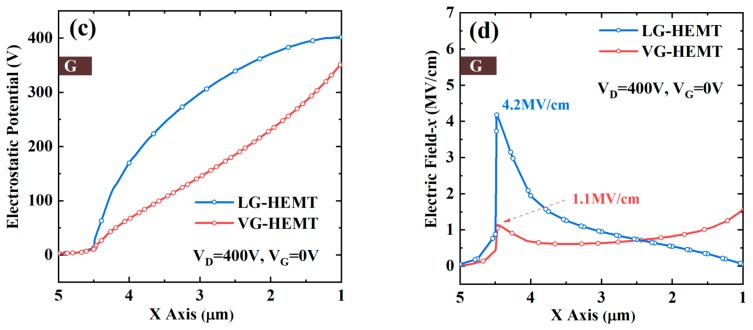
Comparisons of (**a**) electrostatic potential and (**b**) electric field distribution profiles between the LG-HEMT and VG-HEMT devices. The data plots in (**c**) and (**d**) are derived along the two-dimensional electron gas (2DEG) channel within 5 nm near the gate toward the drain side.

Figure 5 shows the 2-D equipotential lines and electric field distributions near the gate region in the same dimension range of the devices at V_D_ = 400 V and V_G_ = 0 V. With comparison, the VG-HEMT exhibits more uniform electrostatic potential and electric field distribution at the gate corner toward the drain side where usually the electrical breakdown occurs due to the high field crowding. Without an additional field plate design which requires more lithography and metal deposition steps, the VG-HEMT can get a reduced electric field at the gate corner owing to the presence of a “natural field plate”, forming by the overlap of gate metal at the etched edge. The depletion region in the vertical gate structure is expanded to a wider range to sustain a higher voltage and hence reduce the device leakage current. Figure 5c,d show the detailed data plots of the electrostatic potential and electric field extracted in the 2DEG channel of both devices. The electrostatic potential increases more smoothly at the gate corner of the VG-HEMT, while a large field crowding happens in the LG-HEMT. The peak of electric field in the VG-HEMT is reduced to 26% compared with the LG-HEMT case, which indicates a better stability and reliability in the VG-HEMT. Furthermore, an improved breakdown voltage can be hopefully achieved when the length of the “natural field plate” is further optimized. To avoid a punch-through breakdown in the VG-HEMT due to the off-state conducting path formed in GaN bulk, carbon doping in GaN bulk to reduce the background carrier density and design of drain-side field plate will be considered and demonstrated in future work.

## 5. Conclusion

In summary, an innovative scheme with a vertical short gate structure in GaN-based HEMTs to realize normally-off operation is proposed and verified by TCAD simulations. The VG-HEMT exhibits a large V_th_ of 3.1 V and an improved output current density and R_on_, which can greatly reduce the power loss of the device. Furthermore, the VG-HEMT displays a lower leakage current during the off state owing to the more uniform electric field distribution by reducing the peak electric field to 26% of the LG-HEMT case. The quantitative dependence of saturated current density and R_on_ on the scattering factors in the trenched vertical gate channel was investigated in detail which concludes the benefit of the proposed short vertical gate VG-HEMT scheme. Although the fabrication process of the VG-HEMT might be complicated and time-consuming, the device structure is proposed for the first time and does have several advantages. More improved performances will be achieved and demonstrated in the following experiments.

## Figures and Tables

**Table 1:** Physical parameters used in technology computer-aided design (TCAD) simulations after calibration [3].

Physical Parameters	Values
Electron effective mass in GaN	0.22·*m_e_*
Electron affinity	3.4 eV
Relative dielectric constant in GaN	9.7
Background electron concentration in i-GaN layer	5.0 × 10^15^ cm^−3^
Electron mobility in 2DEG channel	1500 cm^2^/(V·s)
2DEG sheet density	8.0 × 10^12^ cm^−2^
Electron saturation velocity in GaN	1.8 × 10^7^ cm/s

## References

[1] Fukushima H., Usami S., Ogura M., Ando Y., Tanaka A., Deki M., Kushimoto M., Nitta S., Honda Y., Amano H. (2019). Vertical GaN pn diode with deeply etched mesa and capability of avalanche breakdown. Appl. Phys. Express.

[2] Ando Y., Kaneki S., Hashizume T. (2019). Improved operation stability of Al2O3/AlGaN/GaN MOS high-electron-mobility transistors grown on GaN substrates. Appl. Phys. Express.

[3] Huang H., Liang Y.C., Samudra G.S., Chang T.F., Huang C.F. (2013). Effects of gate field plates on the surface state related current collapse in AlGaN/GaN HEMTs. IEEE Trans. Power Electron..

[4] Chen K.J., Häberlen O., Lidow A., lin Tsai C., Ueda T., Uemoto Y., Wu Y. (2017). GaN-on-Si power technology: Devices and applications. IEEE Trans. Electron Devices.

[5] Huang H., Sun Z., Cao Y., Li F., Zhang F., Wen Z., Zhang Z., Liang Y.C., Hu L. (2018). Investigation of surface traps-induced current collapse phenomenon in AlGaN/GaN high electron mobility transistors with schottky gate structures. J. Phys. D Appl. Phys..

[6] Wei J., Lei J., Tang X., Li B., Liu S., Chen K.J. (2017). Channel-to-channel coupling in normally-off GaN double-channel MOS-HEMT. IEEE Electron Device Lett..

[7] Rossetto I., Meneghini M., De Santi C., Pandey S., Gajda M., Hurkx G.M., Croon J., Šonský J., Meneghesso G. (2018). 2DEG retraction and potential distribution of GaN–on–Si HEMTs investigated through a floating gate terminal. IEEE Trans. Electron Devices.

[8] Wang H., Wei J., Xie R., Liu C., Tang G., Chen K.J. (2016). Maximizing the performance of 650-V p-GaN gate HEMTs: Dynamic RON characterization and circuit design considerations. IEEE Trans. Power Electron..

[9] Sun R., Liang Y.C., Yeo Y.C., Zhao C. (2017). Au-Free AlGaN/GaN MIS-HEMTs With Embedded Current Sensing Structure for Power Switching Applications. IEEE Trans. Electron Devices.

[10] Shen F., Hao R., Song L., Chen F., Yu G., Zhang X., Fan Y., Lin F., Cai Y., Zhang B. (2019). Enhancement mode AlGaN/GaN HEMTs by fluorine ion thermal diffusion with high V th stability. Appl. Phys. Express.

[11] Wu T.L., Marcon D., You S., Posthuma N., Bakeroot B., Stoffels S., Van Hove M., Groeseneken G., Decoutere S. (2015). Forward bias gate breakdown mechanism in enhancement-mode p-GaN gate AlGaN/GaN high-electron mobility transistors. IEEE Electron Device Lett..

[12] Wang H., Wang J., Li M., Cao Q., Yu M., He Y., Wu W. (2018). 823-mA/mm Drain Current Density and 945-MW/cm 2 Baliga’s Figure-of-Merit Enhancement-Mode GaN MISFETs With a Novel PEALD-AlN/LPCVD-Si 3 N 4 Dual-Gate Dielectric. IEEE Electron Device Lett..

[13] Wang Y.H., Liang Y.C., Samudra G.S., Huang H., Huang B.J., Huang S.H., Chang T.F., Huang C.F., Kuo W.H., Lo G.Q. (2015). 6.5 V high threshold voltage AlGaN/GaN power metal-insulator-semiconductor high electron mobility transistor using multilayer fluorinated gate stack. IEEE Electron Device Lett..

[14] Huang H., Liang Y.C. (2015). Formation of combined partially recessed and multiple fluorinated-dielectric layers gate structures for high threshold voltage GaN-based HEMT power devices. Solid-State Electron..

[15] Huang X., Liu Z., Lee F.C., Li Q. (2014). Characterization and enhancement of high-voltage cascode GaN devices. IEEE Trans. Electron Devices..

[16] Ren J., Tang C.W., Feng H., Jiang H., Yang W., Zhou X., Lau K.M., Sin J.K. (2018). A Novel 700 V Monolithically Integrated Si-GaN Cascoded Field Effect Transistor. IEEE Electron Device Lett..

[17] Oka T., Nozawa T. (2008). AlGaN/GaN recessed MIS-gate HFET with high-threshold-voltage normally-off operation for power electronics applications. IEEE Electron Device Lett..

[18] Li X., Hove M.V., Zhao M., Geens K., Lempinen V.P., Sormunen J., Groeseneken G., Decoutere S. (2017). 200 V Enhancement-Mode p-GaN HEMTs Fabricated on 200 mm GaN-on-SOI with Trench Isolation for Monolithic Integration. IEEE Electron Device Lett..

[19] Ma J., Matioli E. (2017). High performance tri-gate GaN power MOSHEMTs on silicon substrate. IEEE Electron Device Lett..

[20] Zhang Y., Sun M., Piedra D., Hu J., Liu Z., Lin Y., Gao X., Shepard K., Palacios T. 1200 V GaN vertical fin power field-effect transistors. Proceedings of the 2017 IEEE International Electron Devices Meeting (IEDM).

[21] Nishimura T., Kasai T., Mishima T., Kuriyama K., Nakamura T. (2019). Reduction in contact resistance and structural evaluation of Al/Ti electrodes on Si-implanted GaN. NUCL INSTRUM METHODS PHYS RES B.

[22] Arora N.D., Hauser J.R., Roulston D.J. (1982). Electron and hole mobilities in silicon as a function of concentration and temperature. IEEE Trans. Electron Devices.

[23] Barnes J.J., Lomax R.J., Haddad G.I. (1976). Finite-element simulation of GaAs MESFET’s with lateral doping profiles and submicron gates. IEEE Trans. Electron Devices.

[24] Lombardi C., Manzini S., Saporito A., Vanzi M. (1988). A physically based mobility model for numerical simulation of nonplanar devices. IEEE Trans. Comput. Aided Des. Integr. Circuits Syst..

[25] Zhang Y., Sun M., Joglekar S.J., Fujishima T., Palacios T. (2013). Threshold voltage control by gate oxide thickness in fluorinated GaN metal-oxide-semiconductor high-electron-mobility transistors. Appl. Phys. Lett..

[26] Hua M., Zhang Z., Wei J., Lei J., Tang G., Fu K., Cai Y., Zhang B., Chen K.J. Integration of LPCVD-SiN x gate dielectric with recessed-gate E-mode GaN MIS-FETs: Toward high performance, high stability and long TDDB lifetime. Proceedings of the 2016 IEEE International Electron Devices Meeting (IEDM).

[27] Yang C., Luo X., Zhang A., Deng S., Ouyang D., Peng F., Wei J., Zhang B., Li Z. (2018). AlGaN/GaN MIS-HEMT with AlN interface protection layer and trench termination structure. IEEE Trans. Electron Devices.

[28] Hao R., Li W., Fu K., Yu G., Song L., Yuan J., Li J., Deng X., Zhang X., Zhou Q. (2017). Breakdown enhancement and current collapse suppression by high-resistivity GaN cap layer in normally-off AlGaN/GaN HEMTs. IEEE Electron Device Lett..

[29] Hu Q., Li S., Li T., Wang X., Li X., Wu Y. (2018). Channel Engineering of Normally-OFF AlGaN/GaN MOS-HEMTs by Atomic Layer Etching and High-k Dielectric. IEEE Electron Device Lett..

[30] Huang H., Liang Y.C., Samudra G.S., Ngo C.L. (2014). Au-free normally-off AlGaN/GaN-on-Si MIS-HEMTs using combined partially recessed and fluorinated trap-charge gate structures. IEEE Electron Device Lett..

[31] Lin S., Wang M., Sang F., Tao M., Wen C.P., Xie B., Yu M., Wang J., Hao Y., Wu W. (2016). A GaN HEMT structure allowing self-terminated, plasma-free etching for high-uniformity, high-mobility enhancement-mode devices. IEEE Electron Device Lett..

[32] Zhang Z., Fu K., Deng X., Zhang X., Fan Y., Sun S., Song L., Xing Z., Huang W., Yu G. (2015). Normally Off AlGaN/GaN MIS-high-electron mobility transistors fabricated by using low pressure chemical vapor deposition Si 3 N 4 gate dielectric and standard fluorine ion implantation. IEEE Electron Device Lett..

[33] Zhang Z., Li W., Fu K., Yu G., Zhang X., Zhao Y., Sun S., Song L., Deng X., Xing Z. (2016). AlGaN/GaN MIS-HEMTs of Very-Low *V* Hysteresis and Current Collapse With In-Situ Pre-Deposition Plasma Nitridation and LPCVD-Si3N4 Gate Insulator. IEEE Electron Device Lett..

[34] Xu Z., Wang J., Liu J., Jin C., Cai Y., Yang Z., Wang M., Yu M., Xie B., Wu W. (2014). Demonstration of normally-off recess-gated AlGaN/GaN MOSFET using GaN cap layer as recess mask. IEEE Electron Device Lett..

[35] Sze S.M., Ng K.K. (2006). Physics of semiconductor devices.

[36] Rodriguez N., Roldan J.B., Gamiz F. (2007). An electron mobility model for ultra-thin gate-oxide MOSFETs including the contribution of remote scattering mechanisms. Semicond. Sci. Technol..

[37] Du J., Yan H., Yin C., Feng Z., Dun S., Yu Q. (2014). Simulation and characterization of millimeter-wave InAlN/GaN high electron mobility transistors using Lombardi mobility model. J. Appl. Phys..

